# A finite element analysis of different postures and intra-abdominal pressures for the uterine ligaments in maintaining the normal position of uterus

**DOI:** 10.1038/s41598-023-32368-z

**Published:** 2023-03-28

**Authors:** Jialan Chen, Junfeng Zhang, Fan Wang

**Affiliations:** 1 Department of Gynecology, Shaanxi Provincial People’s Hospital, Xi’an, 710068 People’s Republic of China; 2 School of Mechanical and Electrical Engineering, Xi’an University of Architecture and Technology, Xi’an, 710055 People’s Republic of China

**Keywords:** Computational biology and bioinformatics, Medical research

## Abstract

Uterine prolapse is a common gynecological disease, which seriously affects the quality of life and physical and mental health of elderly women. The aim of this study was to analyze the influence of different conditions (intra-abdominal pressure (IAP) and posture) on stress and displacement of uterine ligaments using the finite element method, and evaluate the contribution of uterine ligaments on uterus. The three-dimensional (3D) models of retroverted uterus and its accessory ligaments were established, loads and constraints were set in ABAQUS software, and the stress and displacement of uterine ligaments were calculated. The uterine displacement increased with the increase of IAP, and then the stress and displacement of each uterine ligament also increased. The uterine displacement was in the order of forward < upright < backward with different postures, and USL, CL and RL got larger values when the body was backward, while BL got a larger value when the body was forward. With the same condition, the stress of the uterine ligaments was in the order of USL > BL > CL > RL, and the displacement of the ligaments was in the order of BL > RL > USL > CL. The contribution of each uterine ligament changing with different IAP and postures was studied through finite element analysis, and the research results were consistent with the clinical data, which can provide a basis for exploring the mechanism of uterine prolapse.

## Introduction

Uterine prolapse (UP) is a common gynecological disease, which can lead to urination, defecation, sexual dysfunction and pelvic pain. And it seriously affects the quality of life and physical and mental health of elderly women. A survey from Women's Health Initiative shows that the prevalence of mild UP in American women aged 50–79 is 3.8% − 14.2%, and the overall incidence is increasing year by year^1^. The uterus of normal women is mainly affected by the load of intra-abdominal pressure (IAP) and gravity, in which, the gravity direction is vertical downward, and the IAP mainly acts on the uterine corpus. According to the integral theory, the maintenance of uterine physiological position depends on the balance between the support force of supporting tissues (such as ligaments) and the load. Besides, some scholars have found that the change of body tilt angle has a direct impact on IAP^2,3^, that is, the posture has a certain effect on the stress and displacement of uterine ligaments^4^, and then the physiological position of the uterus will be affected. In addition, retroversion of the uterus is common, approximately 1 in 5 women has this condition, while there are few previous studies on this aspect in the published literature. Therefore, it is necessary to study the supporting effect of uterine ligaments in different conditions on the retroverted uterus.

The uterine ligaments are located in the pelvic cavity of human body, and it is still impossible to carry out in vivo non-invasive biomechanical testing in the short term. Although the mechanical test of cadaver tissue can be carried out, while their elastic properties are quite different from those of living tissue. In contrast, the finite element analysis (FEA) can make up for its shortcomings, and it has been widely used in the field of biomechanics research in skeleton^5–7^. However, the application of FEA in the field of obstetrics and gynecology is still in its infancy, which is the hotspot and direction of research. Shan et al.^8^ established a digitalized visible model of the female pelvic of the Chinese visible human. Guo et al.^9^ reconstructed a model of the female pelvic muscles with MR images from 20 women diagnosed with various gynecological diseases by the Mimics software. Furthermore, some scholars have studied the three-dimensional (3D) modeling of uterosacral ligament (USL) and cardinal ligament (CL)^10–14^. Ma et al.^15^ established a finite element model of USL and CL, through which they analyzed the stress distribution and deformation with USL and CL under different values of IAP. Chen^16^ established a relatively complete normal female pelvic three-dimensional finite element model, and she found that BL and USL play a main role in maintaining normal position of the uterus. Rubod^4^ performed a simulation of pelvic mobility with a validated numerical model in a normal situation (standing up to lying down) or induced pathological ones, and they found that uterine ligaments play an important role in pelvic statics. Liu et al.^17^ established a finite element model of the whole pelvic support system (including the uterus, vagina with cavity, CL and USL, levator ani muscle, rectum, bladder, perineal body, pelvis, and obturator internus and coccygeal muscles), the relationship between high IAP and the compliance of the pelvic floor support system in a normal woman without pelvic organ prolapse (POP) was studied on the basis. Gordon et al.^18^ and Bhattarai et al.^19^ established the finite element models of vaginal wall and its supporting tissue, the vaginal prolapse was studied on the basis. Moreover, some scholars used FEA to study the effect of mesh anchoring technique (simple stich and continuous stitch) and the behavior of implants used to replace damaged apical ligaments, such as USL and CL^20,21^.

Based on above researches, this paper establishes finite element models of uterus and its accessory ligaments, then the stress and deformation of the ligaments under different loads and postures are analyzed, and the contributions of uterine ligaments in maintaining the mechanical stability of the uterus are evaluated. This study can provide a basis for exploring the mechanism of UP, and provide reference for the study of biomechanical stability of other POP.

## Methods

The study was approved by the Ethics Committee of Shaanxi Provincial People's Hospital (No. 2022-K120). All procedures were in accordance with the ethical standards of the National Research Committee and with the 1964 Helsinki declaration and its later amendments. All participants provided written informed consent.

### Establishment of 3D geometric model

**Figure 1:**
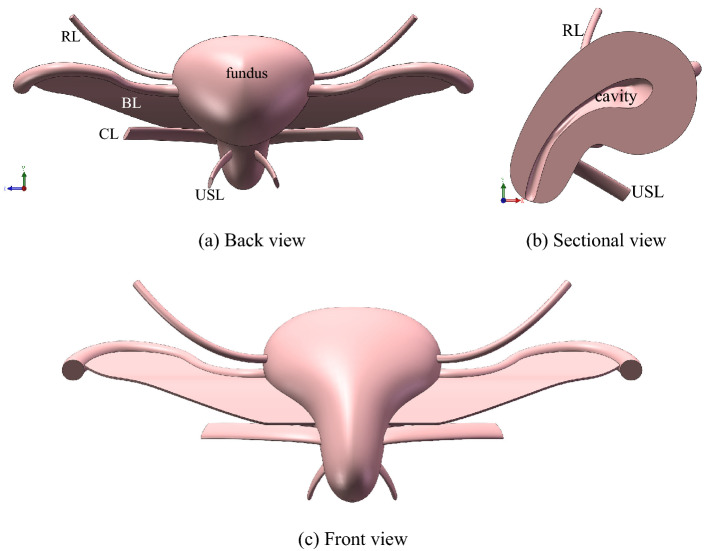
3D model of uterus and its accessory ligaments. **a** Back view, **b** Sectional view. **c** Front view. (created by Solidworks 2018, https://www.solidworks.com/zh-hans).

In this manuscript, the three-dimensional (3D) models of retroverted female uterus and its accessory ligaments were established by SolidWorks software (Fig. 1) based on pelvic MRI images. The MRI images was obtained was in 2020, and the woman was born in1976 without POP and not in menopause. The uterus is a thick-walled muscular organ with a cavity, which is slightly flattened anteriorly and posteriorly in the shape of an inverted pear. The uterine corpus (including cavity) was established based on the MRI images of a female pelvic cavity without UP, and the simplified models of ligaments were obtained with reference to the MRI images and anatomical structures^10,22,23^. The length of uterus from cervix to fundus is 87 mm and the width of cervix is 53 mm, the width of isthmus is 26.5 mm. The broad ligament (BL) is located on both sides of the uterine corpus, broad fan-shaped, the upper end starts from the fallopian tube and the lower end ends in the cervical isthmus with the thickness of 1 mm.

Besides, CL is located at the lower end of BL (rectal side), the angle between the two ligaments is 80º, and the section is oval and the thickness is about 3 mm. USL is located at the lower end of BL and slightly lower than CL, the angle between the two ligaments is 60º, and the section is elliptical with a thickness of about 2 mm. The round ligament (RL) starts from the front of the uterine horn, below the proximal fallopian tube, and extends toward anteroinferior. And its section is round, and length is 70 mm in pelvic cavity with the diameter being of 3 mm. The 3D model was imported into ABAQUS software, then the material characteristics, boundary and load were set. After that, the influence of IAP and posture on the uterus and ligaments were studied.

### Establishment of finite element model

#### Material properties

The material parameters of the 3D model were determined according to the relevant literatures^15,16^. The elastic modulus of the uterus is 2.0 MPa, and its Poisson's ratio is 0.45 with the density being of 1120 kg/m^3^. The elastic modulus of RL, CL, USL and BL are 5.3, 5.54, 8.19 and 4.23 MPa, respectively, and their Poisson's ratio is 0.3.

#### Mesh division

**Figure 2:**
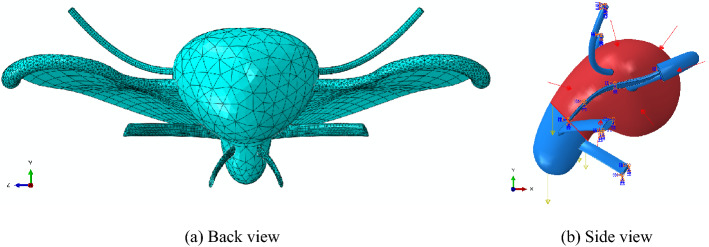
Finite element mesh and boundary conditions. **a** Back view. **b** Side view. (created by Abaqus 2020, https://www.3ds.com/zh/products-services/simulia/products/abaqus/).

The mesh types of uterus and ligaments were determined according to their geometric shapes and the types recommended by ABAQUS software (Fig. 2a). The element sizes controlling mesh density were obtained according to the recommended values of ABAQUS software after appropriate rectification. Ten-node quadratic tetrahedral element (C3D10) was used for the mesh generation of uterus and USL, with the element size being of 4 and 1, the number of elements for uterus and USL was 28,874 and 10,227, respectively. And eight-node linear hexahedral element (C3D8R) was used for the mesh generation of BL, RL and CL, their elements number was 168, 798 and 2436, respectively, with the element size being of 4, 1 and 1. The shape and size of the mesh element were checked by the grid check tool in ABAQUS software. The analysis results showed that there was no wrong element in each part.

#### Boundary conditions and loads

The boundary conditions for the uterine ligaments were an encastre condition (fixed), as shown in Fig. 2b, and the constraints between uterus and the ligaments were binding. Considering that the uterine weight of normal unpregnant women is about 50 g, this study assumed that the gravity of uterus is constant during the study. The load of gravity in ABAQUS software is optional, which is the product of density, component volume and gravity acceleration (9810 mm/s^2^). The gravity direction is determined by three gravity acceleration components (X, Y and Z in turn). Combined with the model coordinate system, the gravity direction of the uterus is always vertical downward (Y direction, yellow arrow in Fig. 2).

For the uterus is an interpositional organ and the cervix is located outside the peritoneum, IAP only acts on the uterine corpus. Therefore, the uterus model was sliced at the cervical isthmus (near the section under BL), and the simulated IAP (red arrow in Fig. 2b) was applied on the surface of the uterus (red surface in Fig. 2b). Referring to the 5th, 50th and 95th percentiles (60, 99 and 168 cm H_2_O, 1 cm H_2_O = 0.098 kPa) of the maximum Valsalva action in DeLancey’s researches^24,25^, the three level of IAP used in this study was 6, 10 and 17 kPa.

**Figure 3:**
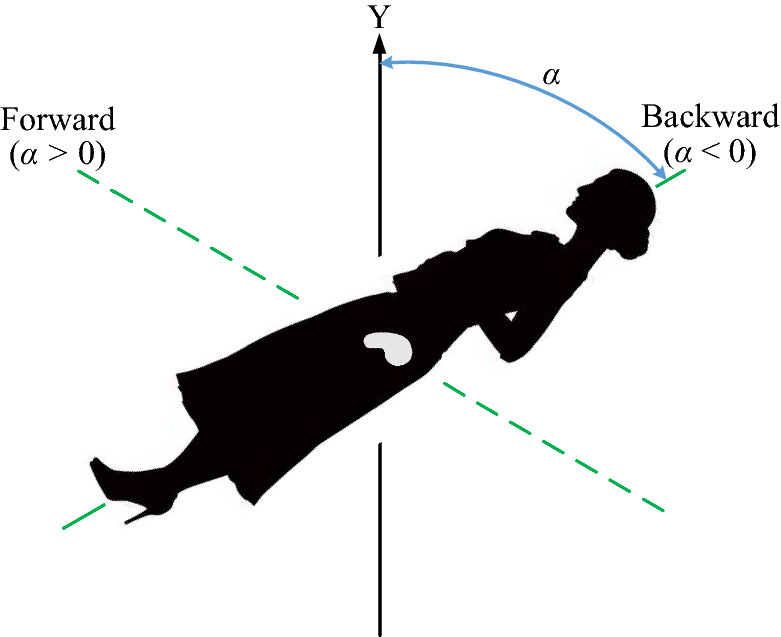
Body tilt angle diagram with different postures.

In order to study the displacement of the uterus and the von Mises stress (stress for short) and displacement of uterine ligaments in different postures, in which the postures are reflected by the tilt angle of α (the angle down from the positive Y-axis). When the body leans forward, the value of α is positive (*α* > 0), while the body is backward, *α* is negative (*α* < 0), and α equals 0º when the body is upright, as shown in Fig. 3. Referring to the tilt angle of the upper body in various sitting or squatting postures in daily life, the angel of α ranges from − 60º to 60º.

## Results

The results presented in this section do not deal with a statistical analysis, but are aimed to show the influence of the changes in specific conditions on the uterus and its accessory ligaments through numerical simulation.

### Simulation of different conditions on uterus

**Figure 4:**
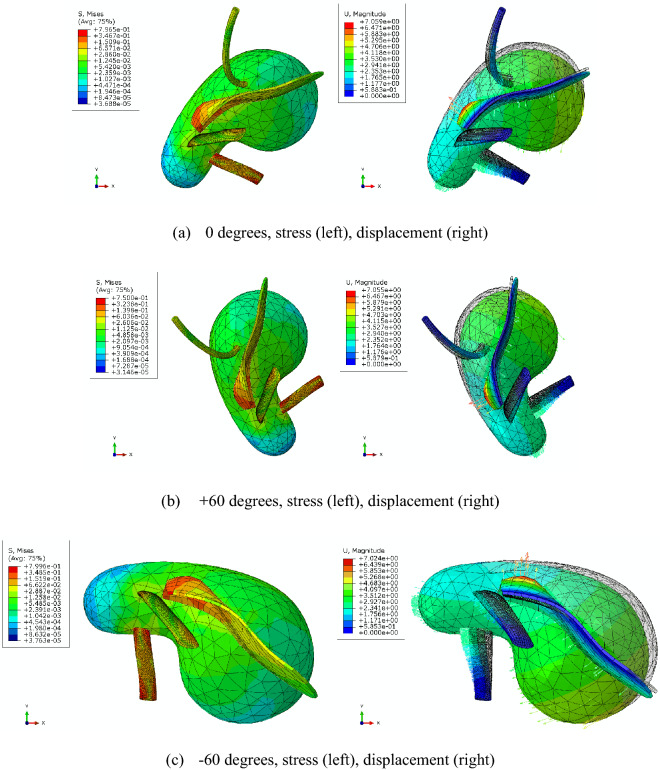
Distribution of displacement and stress in different postures. **a** 0 degrees, stress (left), displacement (right). **b** +60 degrees, stress (left), displacement (right). **c** −60 degrees, stress (left), displacement (right). (IAP = 10 kPa, created by Abaqus 2020, https://www.3ds.com/zh/products-services/simulia/products/abaqus/).

**Figure 5:**
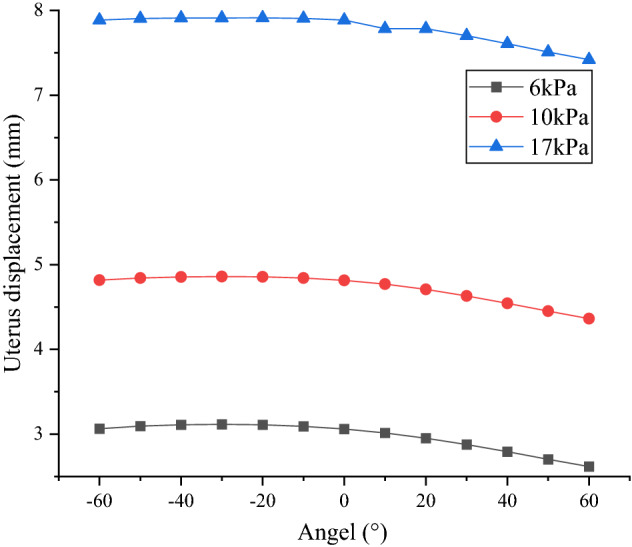
Changes of uterine displacement in different postures (created by OriginPro 2021, https://www.originlab.com/).

The uterus model in this paper is retroverted, its anterior wall extends and the posterior wall bends. Since IAP mainly acts on the circumference of the uterine corpus, the anterior wall was subjected to a posteroinferior force (rectal direction), the posterior wall was subjected to an anterosuperior force, and the fundus was subjected to a resultant force in the direction of cervix. Given the small weight of the uterus, uterine movement was mainly affected by IAP. When the body was upright, forward or backward, the overall motion directions of the uterus and cervix were posteroinferior and anteroinferior (vaginal direction), respectively (Fig. 4). However, the gravity center of uterus was different in different postures, so the size and direction of the resultant force of gravity and IAP on the uterus were different, which in turn had different effects on the magnitude and direction of uterine displacement (Fig. 5).

The maximum displacement of uterus was located at the fundus in different postures, and the displacement leaning back was greater than that of standing upright, while the forward displacement was less than that of upright. When the backward angle increased from small, the uterine displacement increased first and then decreased, and the inflection point was formed at the angel of 30°. Whereas, when the body leaned forward, the greater the anteversion angle, the smaller the uterine displacement. For example, when IAP was 10 kPa, the uterine displacement at 60º was 0.91 times that at 0º, while the uterine displacement at − 30º was the largest and 1.01 times that of 0º. In addition, it can be seen from Fig. 5 that IAP had a direct impact on uterine displacement, which increased with the increase of IAP. For example, the maximum displacement of uterus under 10 kPa was 1.58 times that of under 6 kPa, while it was 0.61 times that of under 17 kPa.

### Simulation of different conditions on uterine ligaments

When the uterus moved under IAP, the displacements of uterine ligaments were not the same due to the relative position difference with the uterus and cervix (Fig. 4). USL and CL were connected to the cervix, both of them were stretched when the cervix moved toward anteroinferior. Their stress and displacement were mainly distributed in the middle and upper segment (near the cervix), and the maximum stress and displacement were located at the junction with the cervix. BL was located between the two sides of uterus and lateral pelvic wall, when the uterus moved toward posteroinferior and the cervix moved toward anteroinferior, its upper segment was stretched and the lower segment was bent. Its stress and displacement were mainly distributed in the middle and lower segment, its maximum stress was concentrated in the lower end of the connection with the pelvic wall, and the maximum displacement was located in the lower part. The end of RL was connected to the lateral pelvic wall, it was also stretched when the uterus moved toward posteroinferior. Its stress and displacement were also mainly distributed in the middle and lower segment, and the maximum stress and displacement were concentrated at the junction with the uterine horn.

**Figure 6:**
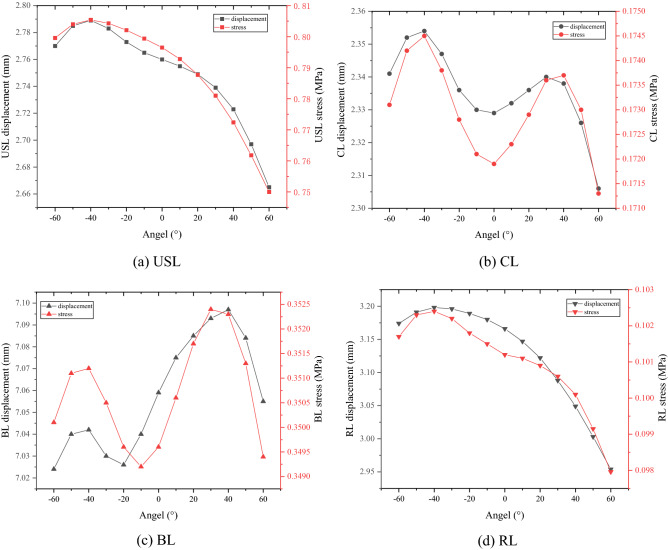
Changes of stress and displacement of uterine ligaments. **a** USL. **b** CL. **c** BL. **d** RL. (IAP = 10 kPa, created by OriginPro 2021, https://www.originlab.com/).

**Figure 7:**
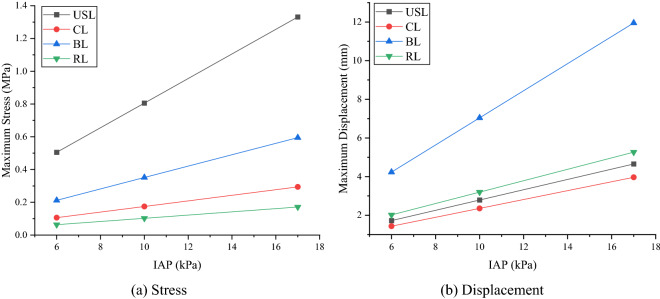
Changes of stress and displacement of ligaments under IAP. **a** Stress. **b** Displacement. (α = − 40º, created by OriginPro 2021, https://www.originlab.com/).

Figure 6 showed that the body posture had a direct impact on the stress and displacement of the uterine ligaments (with IAP being of 10 kPa). In general, when IAP was the same, the stress and displacement of USL and RL in the backward state were greater than those in the forward state. Their stress and displacement were the largest at—40º, then gradually decreased, and the minimum was at 60º. With the vertical state (*α* = 0º) as the center, the stress and displacement of CL first increased and then decreased with the increase of the absolute value of α, and the inflection point formed near the absolute value of 40º. The stress and displacement of BL first increased and then decreased with the gradual increase of forward angle, and the maximum was near α = 40º. With the gradual increase of backward angle, both the values first decreased, then increased and then decreased again, and the inflection point was near α = − 40º. In addition, when the posture was the same (such as − 40º), the stress and displacement of each ligament the stress and displacement of each uterine ligament increased with IAP, as shown in Fig. 7.

In terms of the variation law of stress and displacement of each ligament, generally speaking, USL, CL and RL got larger values when the body was backward, and got the smaller values when the body was forward. Whereas, BL got a larger value when the body was forward, and got a smaller value when the body was backward. Moreover, combining Figs. 6 and 7, it can be seen that, with the same condition, the stress on the ligaments from large to small was USL, BL, CL and RL, and the displacement from large to small was BL, RL, USL and CL. In addition, when the tilt angle changed from − 60º to 60º, the order of maximum-minimum stress difference was USL > RL > CL = BL, and the order of maximum-minimum displacement difference was RL > USL > BL > CL. Accordingly, it can be seen that RL and USL were more sensitive to the change of posture.

## Discussion

UP is one of the common manifestations of POP, which seriously affects women’s health and life quality. Some degree of prolapse is seen in 50% of parous women, and at least 40% of women aged 40–85 years has POP symptoms^26^. The probability of women undergoing surgical treatment for POP in their lifetime is as high as 20%^27^, and the POP treatment requires a lot of medical resources. For example, the annual cost of outpatient medical treatment of pelvic floor diseases in the United States is nearly 300 million US dollars from 2005 to 2006^28^. The present reference method for studying pelvic states and detecting possible pathological conditions remains iconography, unfortunately, most MRI or echography equipment requires patients to lie down. As a result, there is a certain difference between the obtained images and in-body state in daily life. FEA partially allowed this problem to be solved, for which can offer information that is not proposed by conventional methods of investigation such as MRI analysis and anatomical investigations, and it also provides an opportunity to test and/or verify relevant pathophysiology assumptions or research results in the literature^4,29^.

In daily life, standing and squatting are the most common postures other than lying and rest. A research found that the most stretched ligaments are not the same in the standing or lying down, for example, USL supported the most significant stress when standing up, while RL was the most heavily involved when lying down^4^. Accordingly, we can infer that the ligaments with maximum stress and displacement may be different in different postures, which is the starting point of this study. In order to verify the correctness of the above reasoning, this study studied the displacement and stress of uterine ligaments in different postures. According to the maximum IAP during Valsalva maneuver in the literature, three different levels of IAP were determined. And the range of tilt angle was determined according to the common posture in our daily life. It was found that the uterine displacement increased with the increase of IAP, and then the stress and displacement of each uterine ligament also increased. Further, different postures had a direct impact on the displacement and stress of each uterine ligament.

First, the increase of IAP has a direct impact on the uterine displacement. Since the maintenance of the normal position of uterus depends on the dynamic balance between the load (such as IAP) and the support force of supporting tissues (such as ligament), the increase of IAP will affect the above balance. And the continuous increase of the load beyond the limit of ligaments will affect the biomechanical stability of the uterus. With the increase of IAP, the uterine corpus produced a posteroinferior (rectal direction) displacement, while cervix produces an anteroinferior (vaginal direction) displacement, and the downward component was significantly greater than the backward component (uterine corpus) or the forward component (cervix). Moreover, this study found that the uterine displacement increased with the increase of IAP, which was consistent with clinical practice. Related epidemiological studies have shown that long term increase of IAP is a risk factor for POP, which can be caused by chronic constipation, chronic cough and so on^30,31^.

Secondly, posture has a direct impact on uterine displacement. Generally Speaking, when the IAP was constant, the movement direction of the uterus in different postures was posteroinferior, while the movement direction of the cervix was anteroinferior, and the maximum displacement of the uterus was located at the fundus of the uterus. Besides, the uterine displacement in backward state is greater than that of body being upright, while the uterine displacement in forward state is less than that of body being upright. In other words, the posture has a direct impact on the uterine displacement. The possible reason is that the difference in the direction of IAP and gravity, which leads to the different degree of stretching of each uterine ligament, and then the difference of uterine displacement will be caused.

Furthermore, the posture has a direct effect on the stress and displacement of uterine ligaments. According to the integral theory, the cause of UP is the anatomical changes for the injury or relaxation of uterine ligaments^32^. When the IAP was the same, the stress and displacement of USL, CL and RL got larger values when the body leant backward, and the maximum was near − 40º, and this result was consistent with the results of Wang et al.^2^. In other words, the posture has a direct impact on IAP, which in turn affects the stress and displacement of uterine ligaments. Whereas, the stress and displacement of BL got a larger value when the body leant forward, and the maximum was near 40º. Therefore, it can be seen that the UP patients with retroverted uterus should avoid leaning backward as much as possible in their usual posture, especially to avoid leaning back around 40º, so as to reduce the continuous effect of the load on the uterine ligaments. On the contrary, the patients should appropriately increase and keep state of leaning forward.

Besides, when the IAP and posture changed, the stress and displacement of USL and CL were mainly distributed in the middle and upper segment (near the cervix) in different postures, and their maximum stress and displacement were located at the junction with the cervix. And the deformation and stress of BL and RL were mainly distributed in the middle and lower segment, and the maximum stress and displacement were concentrated in the lower part of each ligament. Moreover, when the IAP and posture were the same, the stress of the uterine ligaments was in the order of USL > BL > CL > RL, and the deformation of the ligaments was in the order of BL > RL > USL > CL. Obviously, USL and CL were subjected to greater stress and smaller displacement under the same condition, which further confirmed that USL and CL played an important role in maintaining the biomechanical stability of the uterus^33^.

In addition, this paper established the finite element models of the uterus based on the female pelvic MRI images, and the uterine ligaments were also established according to MRI images and their anatomical structure. So the reliability of the built finite element model was high. Moreover, the gravity of the normal uterus was regarded as a constraint condition for simulation analysis in this study, and the established uterus model contained cavity, which further improved the authenticity of simulation results. Of course, there were certain limitations in this study. For example, only a single uterus model was studied, and the morphology and structure of each ligament were simplified to some extent. The follow-up works will be improved in above aspects to provide more reliable data for clinical research on POP.

In summary, this manuscript used the FEA method to study the influence of IAP and posture on the uterus and ligaments. The study found that the changes of IAP and postures will cause the change of uterine displacement, which in turn lead to the changes of displacement and stress of the uterine ligaments. The results of this study provide a reference for the study of the mechanism of UP and other POP.

## Supplementary Information

Supplementary Information.

## Data Availability

All data analyzed during this study are included in its supplementary information file.
