# Supplementary material for: A finite element analysis of different postures and intra-abdominal pressures for the uterine ligaments in maintaining the normal position of uterus
Source: Sci Rep. 2023 Mar 28;13:5082. doi: 10.1038/s41598-023-32368-z (PMC10050321; doi:10.1038/s41598-023-32368-z)
Supplement: Supplementary file 1 — Supplementary Information. [file 41598_2023_32368_MOESM1_ESM.docx]

| Angel | IAP /kPa | Uterine displacement /mm | Maximum stress /MPa | | | | Maximum displacement /mm | | | |
| --- | --- | --- | --- | --- | --- | --- | --- | --- | --- | --- |
|  |  |  | USL | CL | BL | RL | USL | CL | BL | RL |
| 60 | 6 | 2.617 | 0.4505 | 0.1033 | 0.2107 | 0.05894 | 1.604 | 1.389 | 4.257 | 1.774 |
|  | 10 | 4.363 | 0.750 | 0.1713 | 0.3494 | 0.09796 | 2.665 | 2.306 | 7.055 | 2.954 |
|  | 17 | 7.419 | 1.274 | 0.2903 | 0.5921 | 0.1663 | 4.523 | 3.910 | 11.95 | 5.019 |
| 50 | 6 | 2.703 | 0.4614 | 0.1046 | 0.2121 | 0.05997 | 1.632 | 1.405 | 4.276 | 1.820 |
|  | 10 | 4.451 | 0.7618 | 0.173 | 0.3513 | 0.09915 | 2.697 | 2.326 | 7.084 | 3.003 |
|  | 17 | 7.509 | 1.287 | 0.2927 | 0.5949 | 0.1677 | 4.562 | 3.938 | 12.00 | 5.072 |
| 40 | 6 | 2.792 | 0.4715 | 0.1053 | 0.213 | 0.06081 | 1.656 | 1.415 | 4.286 | 1.864 |
|  | 10 | 4.543 | 0.7724 | 0.1737 | 0.3523 | 0.1001 | 2.723 | 2.338 | 7.097 | 3.049 |
|  | 17 | 7.608 | 1.299 | 0.2935 | 0.5963 | 0.1687 | 4.590 | 3.952 | 12.02 | 5.122 |
| 30 | 6 | 2.876 | 0.4801 | 0.1054 | 0.2132 | 0.06142 | 1.673 | 1.420 | 4.284 | 1.904 |
|  | 10 | 4.631 | 0.781 | 0.1736 | 0.3524 | 0.1006 | 2.739 | 2.340 | 7.093 | 3.088 |
|  | 17 | 7.703 | 1.308 | 0.2929 | 0.5961 | 0.1692 | 4.604 | 3.950 | 12.01 | 5.163 |
| 20 | 6 | 2.952 | 0.4873 | 0.1052 | 0.2129 | 0.06185 | 1.686 | 1.420 | 4.279 | 1.938 |
|  | 10 | 4.709 | 0.7877 | 0.1729 | 0.3517 | 0.1009 | 2.749 | 2.336 | 7.085 | 3.122 |
|  | 17 | 7.785 | 1.314 | 0.2915 | 0.5947 | 0.1692 | 4.609 | 3.940 | 12.00 | 5.194 |
| 10 | 6 | 3.014 | 0.4929 | 0.1049 | 0.2122 | 0.06216 | 1.659 | 1.419 | 4.272 | 1.966 |
|  | 10 | 4.771 | 0.7928 | 0.1723 | 0.3506 | 0.1011 | 2.755 | 2.332 | 7.075 | 3.147 |
|  | 17 | 7.785 | 1.318 | 0.2902 | 0.5927 | 0.1692 | 4.610 | 3.928 | 11.98 | 5.217 |
| 0_ref_ | 6 | 3.060 | 0.4973 | 0.1047 | 0.2116 | 0.06242 | 1.703 | 1.419 | 4.261 | 1.987 |
|  | 10 | 4.815 | 0.7965 | 0.1719 | 0.3496 | 0.1012 | 2.760 | 2.329 | 7.059 | 3.166 |
|  | 17 | 7.887 | 1.320 | 0.2895 | 0.591 | 0.1692 | 4.610 | 3.921 | 11.96 | 5.231 |
| -10 | 6 | 3.092 | 0.5006 | 0.1049 | 0.2113 | 0.06266 | 1.707 | 1.420 | 4.246 | 2.003 |
|  | 10 | 4.842 | 0.7994 | 0.1721 | 0.3492 | 0.1015 | 2.765 | 2.330 | 7.040 | 3.180 |
|  | 17 | 7.907 | 1.322 | 0.2897 | 0.5905 | 0.1694 | 4.614 | 3.921 | 11.93 | 5.241 |
| -20 | 6 | 3.110 | 0.5031 | 0.1053 | 0.2115 | 0.06292 | 1.715 | 1.424 | 4.234 | 2.013 |
|  | 10 | 4.856 | 0.8021 | 0.1728 | 0.3496 | 0.1018 | 2.773 | 2.336 | 7.026 | 3.189 |
|  | 17 | 7.913 | 1.325 | 0.2909 | 0.5914 | 0.1698 | 4.624 | 3.933 | 11.91 | 5.250 |
| -30 | 6 | 3.115 | 0.5048 | 0.1058 | 0.2118 | 0.06316 | 1.721 | 1.430 | 4.232 | 2.019 |
|  | 10 | 4.859 | 0.8043 | 0.1738 | 0.3505 | 0.1022 | 2.783 | 2.347 | 7.030 | 3.196 |
|  | 17 | 7.912 | 1.328 | 0.2927 | 0.5932 | 0.1705 | 4.640 | 3.951 | 11.93 | 5.259 |
| -40 | 6 | 3.110 | 0.505 | 0.1061 | 0.212 | 0.06325 | 1.723 | 1.433 | 4.234 | 2.018 |
|  | 10 | 4.855 | 0.8054 | 0.1745 | 0.3512 | 0.1024 | 2.789 | 2.354 | 7.042 | 3.198 |
|  | 17 | 7.911 | 1.331 | 0.2941 | 0.5948 | 0.171 | 4.653 | 3.966 | 11.95 | 5.265 |
| -50 | 6 | 3.094 | 0.5031 | 0.1058 | 0.2117 | 0.06306 | 1.718 | 1.430 | 4.229 | 2.009 |
|  | 10 | 4.843 | 0.804 | 0.1742 | 0.3511 | 0.1023 | 2.785 | 2.352 | 7.040 | 3.191 |
|  | 17 | 7.905 | 1.331 | 0.294 | 0.595 | 0.171 | 4.652 | 3.966 | 11.96 | 5.263 |
| -60 | 6 | 3.064 | 0.4987 | 0.1049 | 0.2108 | 0.06256 | 1.705 | 1.421 | 4.215 | 1.991 |
|  | 10 | 4.817 | 0.7996 | 0.1731 | 0.3501 | 0.1017 | 2.770 | 2.341 | 7.024 | 3.174 |
|  | 17 | 7.886 | 1.326 | 0.2924 | 0.5938 | 0.1703 | 4.636 | 3.951 | 11.94 | 5.247 |
